# Economic and clinical outcomes among patients with cholangiocarcinoma receiving pemigatinib with or without history of cancer of unknown primary

**DOI:** 10.1093/oncolo/oyaf352

**Published:** 2025-11-08

**Authors:** Sumit Verma, Alejandro Hughes, Nicole M Engel-Nitz, Christina Steiger, Shreekant Parasuraman, Milind Javle, Sunyoung S Lee, Fen Saj, Michael Blecker

**Affiliations:** Incyte Corp., Wilmington, DE, United States; Optum, Inc., Eden Prairie, MN, United States; Optum, Inc., Eden Prairie, MN, United States; Optum, Inc., Eden Prairie, MN, United States; Incyte Corp., Wilmington, DE, United States; Division of Cancer Medicine, University of Texas MD Anderson Cancer Center, Houston, TX, United States; Division of Cancer Medicine, University of Texas MD Anderson Cancer Center, Houston, TX, United States; Division of Cancer Medicine, University of Texas MD Anderson Cancer Center, Houston, TX, United States; Incyte Corp., Wilmington, DE, United States

**Keywords:** biliary tract cancer, cancer of unknown primary, cholangiocarcinoma, health care resource utilization, health care costs, FGFR

## Abstract

**Background:**

There is limited evidence regarding the economic burden, treatment patterns, and overall survival (OS) of patients with cholangiocarcinoma (CCA) and cancer of unknown primary (CUP) who initiated the *FGFR* inhibitor pemigatinib.

**Patients and Methods:**

We used the Komodo Healthcare Map to identify patients with CCA who initiated pemigatinib between 4/17/2020 and 5/31/2023. Follow-up began at initiation and lasted ≥ 1 month. Outcomes included health care resource utilization (HCRU), costs, treatment patterns, and OS.

**Results:**

Two hundred twenty-one patients were included: 78 patients (35.3%) with CUP (median follow-up, 5.9 months) and 143 patients (64.7%) without CUP (median follow-up, 7.3 months). Pemigatinib was similarly well-tolerated in CUP vs non-CUP. Discontinuation was observed in 43.6% vs 49.0% (*P *= .445). Medication possession ratio ≥ 0.80 was achieved by 71.6% vs 67.2% (*P *= .504). CUP was associated with significantly higher prevalence of metastatic disease (100.0% vs 63.6%), per patient per month (PPPM) ambulatory HCRU (8.2 vs 5.5), and ambulatory costs ($8584 vs $5308). Medical costs averaged $13 444 vs $9881 PPPM for CUP and non-CUP, respectively (*P *= .066). Median OS was significantly shorter with CUP (10.2 vs 30.7 months).

**Conclusion:**

Although pemigatinib was similarly well-tolerated regardless of CUP status, patients with CUP incurred greater ambulatory burden and had poorer OS. Patients with CUP were more likely to have evidence of metastatic disease at pemigatinib initiation, which may help explain these results. With the advent of targeted treatments for gene-altered CCA, reflexive genomic testing should be encouraged for all patients with CUP.

## Graphical Abstract

**Graphical Abstract:**
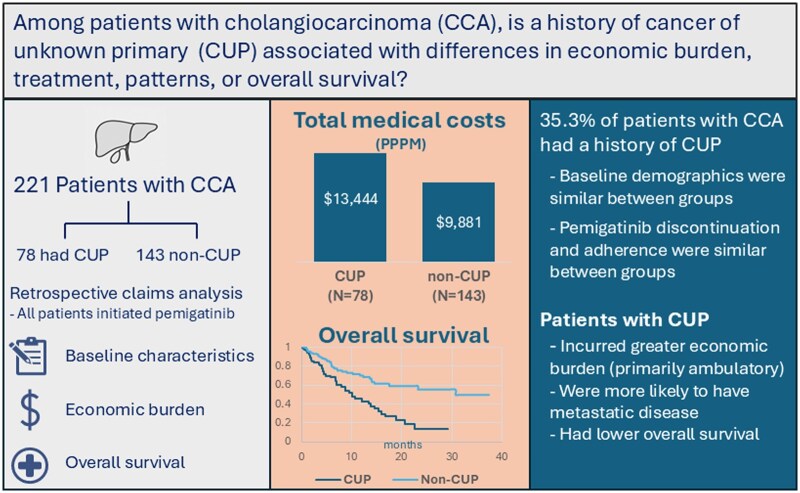

Implications for Practice Among patients with cholangiocarcinoma (CCA), a prior diagnosis of cancer of unknown primary (CUP) is associated with poorer outcomes, including lower overall survival (OS). This retrospective study examined economic burden, treatment patterns, and OS by CUP status among 221 patients with CCA who initiated pemigatinib. Of these, 35.3% had CUP. These patients showed similar medication discontinuation and adherence but worse OS and higher economic burden, particularly in ambulatory care. Patients with CUP may have had more advanced disease at pemigatinib initiation. Reflexive genomic testing upon CUP diagnosis may help reduce delays in identifying actionable targets and initiating appropriate targeted therapy.

## Introduction

Cholangiocarcinoma (CCA) is a malignancy of the biliary tract characterized by heterogeneous and aggressive tumors.^1^^,^^2^ Approximately 10%-15% of primary liver cancers and 3% of all gastrointestinal cancers are CCA.^2–5^ The incidence of CCA increased worldwide over recent decades,^1^^,^^2^^,^^6–8^ including a 43.8% rise in the US between 2001 and 2017.^2^ Diagnosis of CCA often occurs at advanced or metastatic stages of disease, leading to poor prognosis.^1^^,^^9^^,^^10^ Median overall survival (OS) in the US ranged from approximately four to nine months between 1973 and 2017.^2^^,^^4^

The nonspecific clinical presentation, absence of distinguishable localizing symptoms, and lack of definitive pathologic diagnosis—combined with the typically late diagnosis of CCA—can lead to liver-dominant carcinomas being misclassified as cancers of unknown primary (CUP) origin.^2^^,^^5^^,^^11^^,^^12^ Approximately 3%-5% of all cancer diagnoses are CUP, which includes confirmed metastasis absent a definitive primary tumor site.^13^^,^^14^ Common CUP histologies include adenocarcinoma (∼60%), poorly differentiated carcinoma (∼30%), and approximately 5% each of squamous or neuroendocrine carcinoma.^15^ Additional challenges to differentiating CUP tumors include a lack of definitive immunohistochemical markers to identify potential target proteins.^11^ Clinically, approximately 20% of patients with CUP respond to chemotherapy and have a favorable prognosis.^16^ However, the vast majority of cases (approximately 80%) do not respond to chemotherapy. Therefore, patients with CUP often face protracted diagnostic journeys, rapidly spreading metastases, and median OS ranging from two to eight months.^2^^,^^13^^,^^17^

Over the past decade, improved diagnostic practices have helped assign the primary tumor types.^18^^,^^19^ The incidence of cancers counted as liver-involved CUP declined by 54.4% in the US between 2001 and 2017.^2^^,^^18^ However, patients with CCA who have a history of CUP continue to have poorer outcomes overall.^2^^,^^11^^,^^20^ While the proportion of patients with CUP who ultimately have CCA is not well-defined, one study determined that 33.3% of patients suspected of liver-involved CUP met the radiological criteria for intrahepatic CCA (iCCA), yet their survival remained poor, even after reclassification.^11^

Among the many efforts to improve prognoses of both CCA and CUP, continued interest surrounds identifying targetable biomarkers and approved treatments. For CUP, alterations in *KRAS, IDH 1/2, BRCA 1/2*, and *BRAF* were present in 19%-32% of biopsy samples;^21^^,^^22^ at least 24.6% of these patients would be eligible for approved targeted treatments.^21^ Gene alterations are also common with CCA, where an important treatment target includes *FGFR2* fusions and rearrangements.^23^^,^^24^ These alterations are present in approximately 7.5% of CUP cases classified as unfavorable, ie, not responsive to chemotherapy.^16^^,^^22^ In the FIGHT-202 trial (ClinicalTrials.gov identifier: NCT02924376), the *FGFR* inhibitor pemigatinib improved median OS from 7.0 to 17.5 months among patients in the *FGFR2*-altered CCA cohort.^25^ Subsequent real-world evidence also demonstrated pemigatinib’s efficacy as a second-line treatment.^26^^,^^27^ However, the tolerability—measured by discontinuation rate and medication possession ratio (MPR)—of pemigatinib and clinical outcomes among patients with CUP remains unclear.

Although real-world outcomes associated with pemigatinib have been described previously,^26^^,^^27^ there are limited data specifically comparing patients with and without a history of CUP. Characterizing patients by demographics, clinical profile, economic burden, and treatment patterns may fill knowledge gaps and ultimately improve outcomes. Accordingly, this study assessed patients who initiated pemigatinib with three primary objectives:

1. Demographic and clinical comparison: Assess differences in baseline characteristics among patients with CCA, by CUP status (evidence of CUP vs non-CUP).
2. Health care resource utilization (HCRU) and costs: Compare the resource use and associated costs between the two groups.
3. Treatment patterns and outcomes: Analyze differences in treatment sequences before and after initiation of pemigatinib, as well as post-initiation OS.

These findings may help inform providers, payers, and researchers about the unique challenges and unmet needs of CUP patients who may benefit from targeted treatments.

## Materials and methods

### Data source and study design

This was a retrospective analysis of administrative claims from the Komodo Healthcare Map, which includes patients who received medical and pharmacy benefits under commercial, Medicaid, and Medicare Advantage programs. All data were de-identified and remained fully compliant with the Health Insurance Portability and Accountability Act.

**Figure 1.**
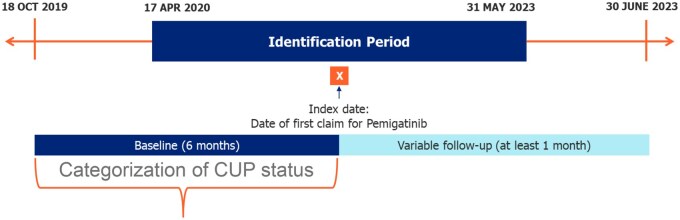
Study time periods. *CUP*, cancer of unknown primary.

The study period lasted from October 18, 2019, through June 30, 2023 (Figure 1). The patient identification period for study eligibility lasted from April 17, 2020, through May 31, 2023. The index date was defined as the date of the earliest claim for pemigatinib (treatment initiation). Baseline was the six months prior to the index date. Follow-up began on the index date and continued for at least one month until the earliest of death, evidence of clinical trial participation, disenrollment from participating insurance coverage, or the end of the study period. Thus, the earliest a patient was eligible for inclusion in the study was April 17, 2020 (with a baseline period beginning October 18, 2019) and the latest a patient was eligible for study inclusion was May 31, 2023, so long as at least six months of baseline and one month of follow-up data were available. In time-to-event analyses, patients were censored if their follow-up ended prior to the occurrence of the event of interest.

### Study population

**Figure 2.**
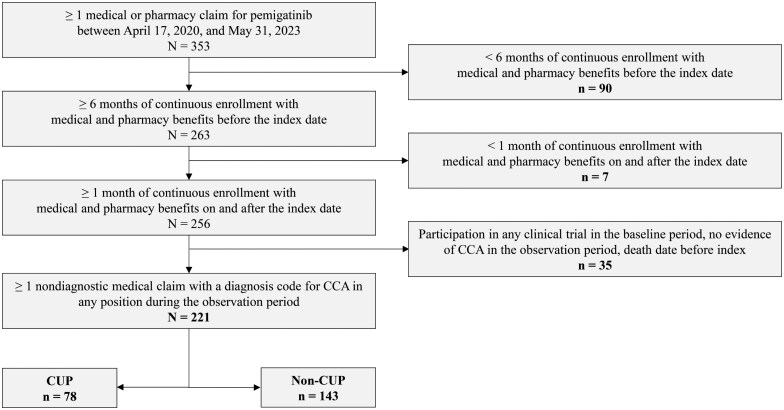
Patient identification and attrition. *CCA*, cholangiocarcinoma; *CUP*, cancer of unknown primary.

The study population consisted of adults aged at least 18 years with at least one pharmacy or medical claim for pemigatinib during the identification period. Additional eligibility criteria are detailed in Figure 2, including: at least six months of continuous enrollment (CE) in participating medical and pharmacy benefits during baseline; at least one month of CE during follow-up; and at least one diagnosis code for CCA (International Classification of Diseases, 10th revision, Clinical Modification [ICD-10-CM]) in any position on a medical claim during baseline or follow-up. Supplementary Table S1 contains the specific ICD-10-CM codes applied for patient selection. Patients with a recorded date of death or any evidence of clinical trial participation during baseline were excluded.

### Measurement of variables and outcomes

Baseline demographic characteristics included age at index year; sex; self-reported race/ethnicity (non-Hispanic White, non-Hispanic Black, Asian or Pacific Islander, Hispanic, or Missing/Other); insurance type (commercial, Medicaid, Medicare Advantage); and geographic region (Northeast, Midwest, South, West/Other, according to the US Census Bureau’s regional designations). Baseline clinical characteristics included the National Cancer Institute (NCI)-adjusted Charlson comorbidity index (CCI) score;^28^ evidence of clinicogenomic testing, eg, *FGFR2* alterations; the presence and site(s) of metastases; the presence and site(s) of other cancers; the site(s) of CCA (iCCA, extrahepatic [eCCA], and/or undifferentiated [code list available in Supplementary Table S1]); and the time between index and CCA diagnosis.

A patient’s CUP status was determined by the presence of any of the following ICD-10-CM codes in any position during baseline: C80.0 (disseminated malignant neoplasm, unspecified), C80.1 (malignant [primary] neoplasm, unspecified), or C80.2 (malignant neoplasm associated with transplanted organ). Among patients diagnosed with CUP, the time from earliest CUP diagnosis to CCA diagnosis was assessed.

Economic outcomes included all-cause HCRU (ambulatory visits [office and hospital outpatient], emergency room [ER] visits, inpatient [IP] admissions, IP days, and pharmacy fills); and all-cause costs (ambulatory, ER, IP, and other medical costs) adjusted to 2022 US dollars according to the medical component of the Consumer Price Index.^29^

Treatment patterns were assessed using medical and pharmacy claims, which include drug name, National Drug Code, dosage, dosage form, and fill date. Medication variables included evidence of baseline anticancer medications (eg, gemcitabine, cisplatin, folinic acid, fluorouracil, oxaliplatin). These medications were classified into baseline treatment regimens based on the claims for the medication(s) received most immediately preceding the index date (treatment date) and in the 28 days immediately preceding the treatment date (treatment window). Treatment regimens were grouped as follows: Folinic acid + Fluorouracil + Oxaliplatin (FOLFOX), gemcitabine + cisplatin (GemCis), GemCis + immunotherapy, None, and Other.

Pemigatinib-related variables included strength of the index date dose; time to the first dose modification during follow-up, if applicable; discontinuation (defined as any gap in medication supply of at least 42 days [two treatment cycles]); time to discontinuation, if applicable; and adherence. Adherence was assessed by MPR, defined as the total number of days of pemigatinib supply (for all but the final fill during the observation period) divided by a patient’s follow-up length, in days.^30^ Claims data cannot confirm medication ingestion, nor can they ascertain the reasons for dose modification or whether treatment modification/discontinuation was due to disease progression. A patient’s date of death was assessed, if applicable.

### Statistical methods

Two-sample *t*-tests (continuous variables, means) and chi-square tests (categorical variables, proportions) compared CUP and non-CUP groups by the baseline demographic characteristics, clinical characteristics, and evidence for anticancer treatment regimens during baseline. Mean all-cause HCRU and costs were converted into per patient per month (PPPM) values and compared by CUP status with 2-sample *t*-tests. Pemigatinib treatment outcomes during follow-up were also compared by CUP status with 2-sample *t*-tests (continuous variables) or chi-square tests (categorical variables). Kaplan–Meier survival analysis estimated mortality and time to discontinuation during follow-up, by CUP status; the log-rank test compared the two groups. All variables and outcomes were analyzed descriptively using SAS 9.4 (Cary, NC). Significance was set at *α* = 0.05 (two-tailed).

### Ethics approval

Institutional review board approval or waiver of approval was not required for this study because the study data were secondary and de-identified in agreement with the United States Department of Health and Human Services Privacy Rule’s requirements for de-identification codified at 45 C.F.R. § 164.514(b).

## Results

### Study population

A total of 221 patients were included and followed for a median (IQR) of 6.9 (3.5-13.8) months. The mean (SD) age was 55.5 (12.3) years and 66.1% were female. Seventy-eight patients (35.3%) were diagnosed with CUP, while 143 (64.7%) were not (Figure 2). Median (IQR) follow-up for patients with and without CUP was 5.9 (2.8-11.6) vs 7.3 (4.0-14.7) months, respectively (*P *< .05). The two groups were similar in baseline age, proportion female, geographic region, insurance type, index year, and NCI-adjusted CCI score (Table 1).

**Table 1.** Baseline demographic and clinical characteristics.

Characteristic	CUP (*n* = 78)	Non-CUP (*n* = 143)	*P*-value
**Age (years), mean (SD)**	54.2 (12.7)	56.1 (12.1)	.264
**Age category (years), *n* (%)**			
** 18-44**	21 (26.9%)	25 (17.5%)	.099
** 45-64**	45 (57.7%)	87 (60.8%)	.649
** 65+**	12 (15.4%)	31 (21.7%)	.259
**Female, *n* (%)**	48 (61.5%)	98 (68.5%)	.294
**Geographical region, *n* (%)**			
** Northeast**	19 (24.4%)	37 (25.9%)	.805
** Midwest**	19 (24.4%)	34 (23.8%)	.923
** South**	21 (26.9%)	47 (32.9%)	.360
** West/Other**	19 (24.4%)	25 (17.5%)	.221
**Race/Ethnicity, *n* (%)**			
** Asian or Pacific Islander or Other**	5 (6.4%)	17 (11.9%)	.194
** Black/African-American**	6 (7.7%)	16 (11.2%)	.407
** Hispanic**	6 (7.7%)	15 (10.5%)	.498
** White**	26 (33.3%)	57 (39.9%)	.338
** Unknown/Missing**	35 (44.9%)	38 (26.6%)	.006
**Insurance type, *n* (%)**			
** Commercial**	54 (69.2%)	83 (58.0%)	.102
** Medicare advantage**	8 (10.3%)	20 (14.0%)	.426
** Medicaid**	9 (11.5%)	29 (20.3%)	.100
** Multiple**	7 (9.0%)	11 (7.7%)	.739
**Index year, *n* (%)**			
** 2020**	21 (26.9%)	31 (21.7%)	.380
** 2021**	28 (35.9%)	47 (32.9%)	.649
** 2022**	17 (21.8%)	40 (28.0%)	.316
** 2023**	12 (15.4%)	25 (17.5%)	.690
**Baseline NCI-adjusted Comorbidity Index, mean (SD)**	1.7 (1.8)	1.6 (1.8)	.714
**Clinicogenomic testing, *n* (%)**	40 (51.3%)	51 (35.7%)	.024
**Evidence of liver cancer, unspecified origin, *n* (%)**	43 (55.1%)	35 (24.5%)	<.001
**Metastatic disease, *n* (%)**	78 (100.0%)	91 (63.6%)	<.001
** Non-lymphatic metastases, excluding liver**	78 (100.0%)	82 (57.3%)	<.001
** Lung**	32 (41.0%)	35 (24.5%)	.011
** Peritoneum**	16 (20.5%)	22 (15.4%)	.334
** Bone**	34 (43.6%)	26 (18.2%)	<.001
** Unknown**	78 (100.0%)	34 (23.8%)	<.001
**CCA location^a^, *n* (%)**			
** Extrahepatic**	14 (18.0%)	20 (14.0%)	.435
** Intrahepatic**	69 (88.5%)	131 (91.6%)	.446
** Undifferentiated**	62 (79.5%)	83 (58.0%)	.001
**Time from CCA diagnosis to index date^b^, mean (SD), months**	4.9 (1.4)	5.3 (1.2)	.032

a A given patient could have more than one CCA location.

b Among patients who had their CCA diagnosis prior to the index date (*n* = 216).

Abbreviations: CCA, cholangiocarcinoma; CUP, cancer of unknown primary; NCI, National Cancer Institute.

The frequencies of metastases and clinicogenomic testing were significantly greater among patients with CUP (all *P *< .05; Table 1). Patients with CUP were also more likely to have an undifferentiated CCA location (*P *< .01), while the proportions of iCCA and eCCA diagnoses were similar between groups. We also assessed the relative timing between CUP and CCA diagnoses, wherein 28 patients with CUP (35.9%) had their CUP diagnosis before CCA. The mean (SD) duration between the two claims was 19.2 (46.3) days. Both CUP and CCA diagnoses were on the same claim for 66 patients (84.6% of patients with CUP). Among these, 36 (54.5%) had CUP as their primary diagnosis on the claim.

### Treatment patterns

Patients diagnosed with CUP were significantly more likely to use GemCis during baseline (prior to pemigatinib initiation), compared with the non-CUP group (*P *< .01; Table 2). The most common initial dose of pemigatinib was 13.5 mg, with no difference by CUP status. Both groups had similar frequencies of dose reduction.

**Table 2.** Medication treatment patterns.

Variable	CUP (*n* = 78)	Non-CUP (*n* = 143)	*P*-value
**Anticancer medication regimens before pemigatinib, *n* (%)**			
** Gemcitabine + Cisplatin (GemCis)**	34 (43.6%)	42 (29.4%)	.033
** Folinic acid + Fluorouracil + Oxaliplatin (FOLFOX)**	7 (9.0%)	10 (7.0%)	.597
**GemCis + immunotherapy**	9 (11.5%)	7 (4.9%)	.069
**Other regimen**	18 (23.1%)	42 (29.4%)	.315
**No evidence of a baseline regimen**	10 (12.8%)	42 (29.4%)	.006
**Starting pemigatinib strength, *n* (%)**			
** 4.5 or 9.0 mg^a^**	9 (11.5%)	20 (14.0%)	.607
** 13.5 mg**	69 (88.5%)	123 (86.0%)	.607
**Strength reductions**			
** First reduction, *n* (%)**	17 (21.8%)	42 (29.4%)	.224
** Time to first reduction, months, mean (SD)**	2.9 (3.4)	3.2 (1.9)	.672
**Discontinuation of index therapy^b^, *n* (%)**	34 (43.6%)	70 (49.0%)	.445
**Reasons for end of follow-up, among those who discontinued, *n* (%)**			
**End of continuous enrollment**	< 15 (< 44.1%)	44 (62.9%)	.037
**Evidence of clinical trial participation**	< 5 (< 14.7%)	6 (8.6%)	.282
**Death**	19 (55.9%)	20 (28.6%)	.007
**End of study period**	0 (0.0%)	0 (0.0%)	N/A
**Time to discontinuation, months, median (IQR)**	4.3 (1.4-6.3)	4.7 (2.9-7.5)	.059
**Medication possession ratio ≥ 0.80**	53 (71.6%)	92 (67.2%)	.504

a Fewer than five patients in both CUP and non-CUP groups initiated pemigatinib at a 4.5-mg dose.

b Discontinuation was defined as any gap in medication of at least 42 days.

Abbreviation: CUP, cancer of unknown primary.

**Figure 3.**
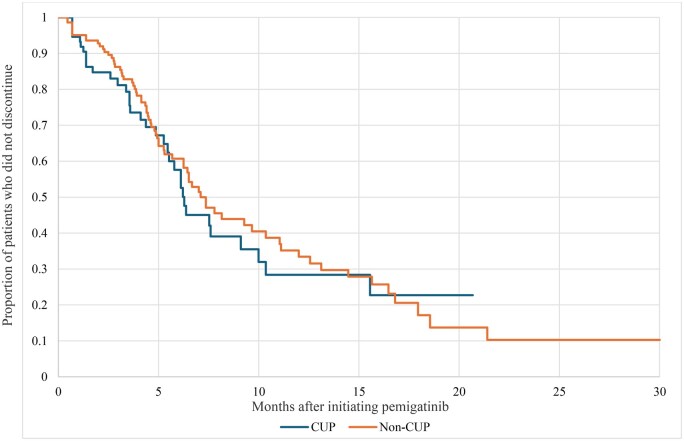
Time to discontinuation^a^ of pemigatinib, by CUP status. ^a^Discontinuation was defined as any gap in medication of at least 42 days. *CUP*, cancer of unknown primary.

Adherence, as measured by proportion with MPR ≥ 0.80, was similar between groups: 71.6% vs 67.2% for patients with and without CUP, respectively (Table 2). Similar rates of discontinuation were also observed: 43.6% vs 49.0% (Table 2 and Figure 3). The corresponding median (IQR) time to discontinuation was 4.3 (1.4-6.3) vs 4.7 (2.9-7.5) months. Patients with CUP were more likely to discontinue due to death (54.9% vs 28.6%, *P *< .01).

### All-cause HCRU and costs

Patients with CUP incurred significantly greater ambulatory and IP visits during baseline, compared with non-CUP (Supplementary Table S2). The mean (SD) PPPM number of ambulatory visits was 7.5 (3.2) vs 5.9 (3.3) for patients with and without CUP, respectively (*P *< .001). The corresponding number of IP stays averaged 0.2 (0.3) vs 0.2 (0.3) PPPM (*P *< .05), while ER utilization was similar between the groups. Total PPPM medical costs during baseline averaged $17 019 ($11 953) vs $13 711 ($14 829) for patients with and without CUP, respectively (*P *= .073). During baseline, only the category of other medical costs (including at-home care or medical devices) was significantly different between groups: $1130 ($1384) vs $669 ($983) (*P *< .05).

**Figure 4.**
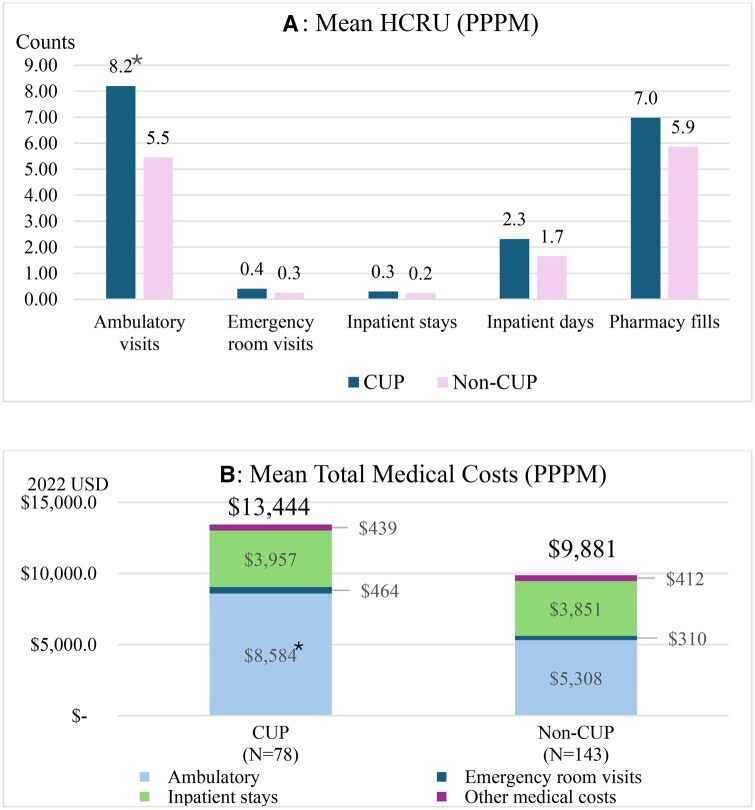
All-cause HCRU and costs (PPPM) during follow-up, by CUP status. *P < .001 compared with non-CUP. Ambulatory HCRU included office and hospital outpatient visits. Medical costs included costs associated with ambulatory, emergency, inpatient, and other medical claims. Abbreviations: *CUP*, cancer of unknown primary; *HCRU*, health care resource utilization; *PPPM*, per patient per month.

During follow-up, ambulatory HCRU and costs were significantly higher among patients with CUP (both *P *< .05; Figure 4A and B; Supplementary Table S2). Other categories of HCRU and costs were similar between groups. Although total medical costs were numerically higher among patients with CUP, the difference was not statistically significant (*P *= .066; Figure 4B).

### Overall survival

**Figure 5.**
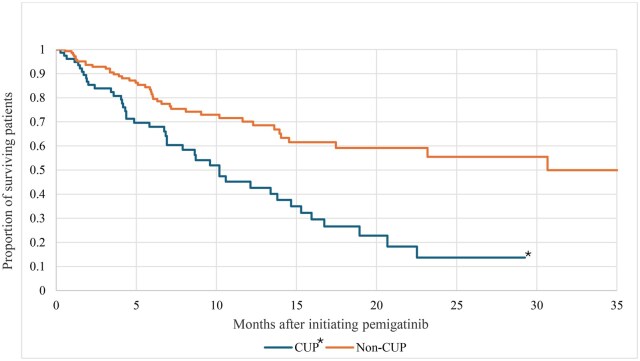
Overall survival during follow-up, by CUP status. *P < .001 compared with non-CUP. *CUP*, cancer of unknown primary.

Kaplan–Meier analysis showed that patients diagnosed with CUP had significantly lower OS during follow-up, compared with non-CUP (Kaplan–Meier median, 10.3 vs 31.1 months; *P *< .001) (Figure 5). The two-year OS was 38.8% for the entire study population: 13.7% vs 55.6% for patients with and without CUP, respectively (*P *< .001).

## Discussion

This was a retrospective claims analysis of 221 patients with CCA who were treated with pemigatinib between April 2020 and May 2023. Our cohort included 78 patients with CUP, as defined by ICD-10-CM codes C80.0-80.2. We determined that CUP was associated with significantly higher ambulatory visits and costs. Patients with CUP had lower OS, despite exhibiting similar pemigatinib treatment patterns. In addition, those with CUP were significantly more likely to have a baseline IP stay, evidence of metastatic disease, GemCis treatment (perhaps indicative of relatively aggressive treatment), clinicogenomic testing, and an undifferentiated CCA location. Our results suggest that pemigatinib was similarly well-tolerated between groups. However, patients with CUP had more advanced disease at treatment initiation and their unmet needs remain substantial.

To our knowledge, this is the first study to compare the clinical features, costs, and treatment patterns in CCA patients treated with pemigatinib who were initially diagnosed with CUP. As many as 32% of CUP cases harbor potentially targetable genetic alterations,^22^ so our results underscore the importance of next-generation sequencing in this setting. Taken in the context of our results with pemigatinib alone, future guidelines should consider reflexive testing for potential molecular targets among patients with CUP. From the payer and administrative perspectives, the increased economic burden (primarily ambulatory) associated with a CUP diagnosis may help set organizational expectations or serve as a basis for future analyses. In addition, payers are encouraged to consider reimbursement for comprehensive genomic testing to identify biomarkers for which targeted therapies exist.

Similar to our findings, prior research determined that CUP was associated with increased economic burden and poorer OS.^11^^,^^13^^,^^17^^,^^20^^,^^31^^,^^32^ The poorer OS observed among patients with CUP is at least partly due to their higher prevalence of metastatic disease. Regarding economic burden, we observed that total medical costs during follow-up averaged $13 444 vs $9881 PPPM for patients with and without CUP, respectively. The difference was primarily driven by ambulatory costs, which averaged $8584 vs $5308. These costs were traced to increased ambulatory visits, which averaged 8.2 vs 5.5 PPPM. The increased ambulatory activity among patients with CUP aligns with the hypothesis that they initiated treatment at a more advanced stage of CCA. While real-world data on the economic burden associated with CUP remain very limited, Gordon et al reported that patients with CUP incurred 47% higher costs than those with ovarian cancer in an Australian cohort.^31^ Like our study, they observed a considerable portion of HCRU involved ambulatory consultations.

Despite differences in economic burden, pemigatinib was similarly well-tolerated between groups. Patients with CUP were apparently more advanced at treatment initiation, yet both groups were similar in proportion of MPR ≥ 0.80, rates of discontinuation, and time to discontinuation. Our results among all patients with CCA align with the modal initial dose (13.5 mg) and discontinuation rate (50.0%) reported by Saverno et al.^26^ Among baseline therapies, GemCis was received by 43.6% and 29.4% of the CUP and non-CUP groups, respectively. This uptake may seem low given that GemCis is viewed as a standard of care.^33^ However, our results are consistent with Healy et al, who reported that 44.6% of patients with biliary tract cancers initiated GemCis as a first-line therapy in the real world.^34^ Notably, their analysis predated the 2022 approval of GemCis plus immunotherapy,^35^ which overlapped with the latter portion of our study period. Our combined proportion of patients with baseline utilization of GemCis and CemCis plus immunotherapy was 55.1% and 34.3% for CUP and non-CUP, respectively. Healey et al also reported FOLFOX utilization by 3.2% and 19.0% of patients as first- and second-line therapies, respectively. In rough agreement, we observed baseline FOLFOX utilization in 9.0% and 7.0% of CUP and non-CUP groups, respectively. Collectively, these observations may reflect the frequent discordance between recommendations and real-world clinical practice. Since we selected for initiation of pemigatinib—a second- or third-line therapy—considerable variability in prior treatment regimens is expected.

This study had several strengths. First, the Komodo Healthcare Map contains a comprehensive pool of de-identified claims data on over 300 million lives sourced from multiple US-based health care plans. This diversity enhances the generalizability of our findings. Second, we reduced the risk of patient misclassification by specifying an index therapy that is recommended as second-line treatment for patients with CCA. These criteria yielded an appropriate comparator group for patients with CUP. Third, our study comes at a time of rapidly evolving diagnostic and treatment options. While the incidence of CUP has decreased over recent years, our results may further inform providers and researchers about the value in thorough diagnosis to reduce the odds of CUP or at least minimize the time between CUP and identification of the primary cancer. Fourth, to our knowledge, we are the first to report on real-world adherence to pemigatinib among patients initially diagnosed with CUP. Finally, this is the first analysis comparing patients with and without CUP who initiated targeted treatment for genetic alterations. Prior studies assessed CUP misclassification or targeted therapies for CCA.^2^^,^^11^^,^^25^^,^^26^ However, the economic burden, treatment adherence, and unmet needs of patients with CUP and CCA remained unexplored to date.

Despite the strength of the data source and specificity of the study population, retrospective analyses contain some inherent limitations. First, claims data do not allow for comprehensive capture of clinicogenomic testing, which has been estimated to occur in 35%-48% of patients with advanced gastrointestinal cancers.^36^^,^^37^ Despite this limitation, the risk of misclassification likely remained low because we restricted the study population to patients with CCA who initiated pemigatinib therapy (which is approved for patients with *FGFR2* alterations). Some patients in our study may have additional alterations for which targeted therapies are available (eg, *IDH 1/2*, *BRAF*, etc.). Second, our HCRU data may not capture all health care encounters. While we excluded patients with evidence of clinical trial enrollment, some patients included in this study may have accessed care outside their plan’s network, including free clinics, sample medications, or unbilled consultations. Third, claims data are not collected for research purposes. We cannot directly confirm disease status, which introduces the possibility of misclassification. However, we expect any misclassification to be non-differential between cohorts and unlikely to systematically bias outcome comparisons. In a related note, generalizability may be affected by the set of ICD-10-CM codes we used to define CUP (C80.0-80.2). While this approach avoided inferring CUP status from metastatic site codes alone, we may have undercounted the true presence of CUP in the study population. Future research could assess definitions of CUP that incorporate a broader set of diagnostic codes. Fourth, we required at least six months of baseline and one month of follow-up CE. Medication discontinuation rates may, therefore, reflect both true discontinuation and administrative censoring (eg, losing insurance coverage). As a result, the observed discontinuation rates may appear higher than expected. The CE requirement may also limit the generalizability of our results to patients with different access to health care, including uninsured patients. Notably, CUP classification was based on the presence of relevant diagnosis codes within this six-month baseline period. The potential remains for left-censoring, whereby CUP diagnoses may have been missed if they occurred outside the baseline window. Finally, the limited follow-up duration may not fully capture longer-term patterns of pemigatinib use.

Our characterization of patients who initiated pemigatinib opens the potential to study the unmet needs of patients with CUP and, ultimately, CCA. Future studies could focus on the timing of the CUP/CCA diagnostic journey by assessing a study population specifically focused on CUP criteria. A larger study population may permit multivariable analyses to better control for factors such as the site of CCA, extent of metastatic disease, and prior lines of therapy. Such endeavors would strengthen understanding of whether a documented history of CUP reflects a distinct, more aggressive disease biology. Second, researchers may consider assessing strategies for minimizing the time between CUP diagnosis and identification of the primary cancer. Topics could include assessing the proportion of CUP cases that harbor *FGFR* or other genetic alterations. Finally, studies could explore the effects of early genetic testing on the timing of targeted treatment initiation and subsequent OS.

## Conclusion

Patients with CUP and CCA were more likely to have metastatic disease prior to pemigatinib initiation, compared with a demographically similar group of patients with CCA but no CUP diagnosis. Despite showing similar treatment patterns, patients with CUP had poorer OS. While our study could not assess the impact of genomic testing on outcomes, our findings underscore the importance of timely diagnosis and molecular characterization in clinical practice. A CUP diagnosis was also associated with increased ambulatory HCRU and costs. Collectively, our results suggest that patients with CUP had more advanced disease upon pemigatinib initiation. Further research is needed to rapidly identify the primary cancer and proportion of patients with genetic alterations so the appropriate targeted therapies can be initiated as early as possible.

## Supplementary Material

oyaf352_Supplementary_Data

## Data Availability

The data contained in the Komodo Healthcare Map contains proprietary elements and, therefore, cannot be broadly disclosed or made publicly available at this time. The disclosure of this data to third-party clients assumes certain data security and privacy protocols are in place and that the third-party client has executed a standard license agreement that includes restrictive covenants governing the use of the data.
