# Supplementary material for: Economic and clinical outcomes among patients with cholangiocarcinoma receiving pemigatinib with or without history of cancer of unknown primary
Source: Oncologist. 2025 Nov 8;30(11):oyaf352. doi: 10.1093/oncolo/oyaf352 (PMC12611303; doi:10.1093/oncolo/oyaf352)
Supplement: oyaf352_Supplementary_Data [file oyaf352_supplementary_data.docx]

**Supplemental Materials**

**Supplemental Table S1.** International Classification of Diseases, 10^th^ revision, Clinical Modification (ICD-10-CM) Codes Used to Identify Patients with any Claim for CCA During the Study Period.

| ICD-10-CM Code | Site | Code Description |
| --- | --- | --- |
| C22.1 | iCCA | Intrahepatic bile duct carcinoma |
| C24.0 | eCCA | Malignant neoplasm of extrahepatic bile duct |
| C24.8 | Unspecified | Malignant neoplasm of overlapping sites of biliary tract |
| C24.9 | Unspecified | Malignant neoplasm of biliary tract, unspecified |
| C78.7 | Unspecified | Secondary malignant neoplasm of liver and intrahepatic bile duct |

Abbreviation: *CCA*, cholangiocarcinoma.

**Supplemental Table S2.** Baseline and Follow-up HCRU and Costs, by CUP status.

| **Outcome (PPPM)** | **Baseline** | | | **Follow-up** | | |
| --- | --- | --- | --- | --- | --- | --- |
|  | CUP  (n = 78) | Non-CUP  (n = 143) | *P*-value | CUP  (n = 78) | Non-CUP  (n = 143) | *P*-value |
| **HCRU**, counts, mean (SD) |  |  |  |  |  |  |
| Ambulatory visits | 7.5 (3.2) | 5.9 (3.3) | < 0.001 | 8.2 (5.7) | 5.5 (3.7) | < 0.001 |
| ER visits | 0.3 (0.3) | 0.2 (0.3) | 0.099 | 0.4 (0.6) | 0.3 (0.5) | 0.051 |
| IP stays | 0.2 (0.3) | 0.2 (0.3) | 0.034 | 0.3 (0.4) | 0.2 (0.4) | 0.366 |
| Pharmacy fills | 4.5 (2.9) | 4.5 (4.0) | 0.911 | 7.0 (4.2) | 5.9 (4.4) | 0.068 |
| **Costs^a^**, USD, mean (SD) |  |  |  |  |  |  |
| Medical | $17,019 ($11,953) | $13,711 ($14,829) | 0.073 | $13,444 ($13,463) | $9,881 ($13,799) | 0.066 |
| Ambulatory | $12,259 ($9,746) | $10,409 ($13,567) | 0.244 | $8,584 ($9,909) | $5,308 ($7,260) | 0.011 |
| ER | $277 ($421) | $252 ($678) | 0.736 | $464 ($726) | $310 ($905) | 0.171 |
| IP | $3,353 ($6,820) | $2,380 ($5,664) | 0.258 | $3,957 ($8,710) | $3,851 ($9,999) | 0.938 |
| Other Medical^b^ | $1,130 ($1,384) | $669 ($983) | 0.010 | $439 ($810) | $412 ($918) | 0.825 |

^a^Inflation-adjusted to USD 2022; ^b^Other medical costs include costs not attributable to ambulatory, ER, or IP claims; examples of other medical costs may include costs for independent laboratory services, home health care, and durable medical equipment. Abbreviations: *CUP*, cancer of unknown primary; *ER*, emergency room; *HCRU*, health care resource utilization; *IP*, inpatient hospitalization; *PPPM*, per patient per month.
